# Hair Color and Hearing Loss: A Survey in a Group of Military Men

**Published:** 2012

**Authors:** Amir Hossain Ghazizadeh, Mehdi Bakhshaee, Ebrahim Mahdavi, Rahman Movahhed

**Affiliations:** 1 *Department of otorhinolaryngology, Shahid Beheshti University of Medical Sciences, Tehran, Iran.*; 2 *Ear, Nose, Throat, Head and Neck surgery Research Center, Mashhad University of Medical Sciences, Mashhad, Iran*; 3 *Audiologist, Shahid Beheshti University of Medical Sciences, Tehran, Iran.*; 4 *Department of otorhinolaryngology, Faculty of Medicine, Mashhad University of Medical Sciences, Mashhad, Iran*

**Keywords:** Hair color, Hearing loss, Noise-induced, Pigmentation, Disease Susceptibility

## Abstract

**Introduction:**

It has been shown that low levels of pigmentation increase susceptibility to noise-induced hearing loss in humans. For this reason, white populations develop more pronounced noise- induced hearing loss in comparison to black populations. Similarly, blue-eyed individuals exhibit greater temporary threshold shift than brown-eyed subjects; still, no strong correlation has been verified between the lightness of hair color and susceptibility to noise-induced hearing loss. This study was performed with the purpose of investigating a possible association between hair color and the degree of hearing loss due to firing noise. Study Design: Prospective observational study. Setting: A tertiary referral center with an accredited otorhinolaryngology-head & neck surgery department.

**Materials and Methods:**

A total of 57 military recruits were divided into two groups; light-colored (blond and light brown) and dark-colored hair (dark brown and black). The two groups were matched based on history of firing noise exposure (number of rounds; type of weapon) and the level of hearing loss at 2, 3, 4, 6 and 8 kHz sound frequencies was compared between them.

**Results:**

The results showed that the mean level of hearing loss of light-colored hair individuals (20.5±17dB) was significantly greater than that of dark-haired subjects (13.5±11dB), (P=0.023).

**Conclusion:**

The results indicate that hair color (blond versus black) can be used as an index for predicting susceptibility to noise-induced hearing loss in military environments. Therefore, based on the individual's hair color, upgraded hearing conservation programs are highly recommended.

## Introduction

Today we are all surrounded by a very noisy environment. Beside presbycosis (age-related hearing loss), the most common type of sensorineural hearing loss is related to high-noise environments. Noise-induced hearing loss is a major problem in industrialized countries and, due to the high rate of industrialization in developing countries and the lack of adequate protective equipment, this disorder is ever increasing in prevalence (1). Studies conducted on noise-induced hearing loss have mainly focused on reduction in hearing sensitivity; whereas exposure to noise has other numerous effects on the hearing system that could even affect the perception of voice. 

Excessive noise exposure is annoying, produces stress, impairs communication, interferes with work and leisure time activities, and in high enough doses, produces permanent damage to the auditory system which leads to significant hearing loss (2). Occupational noise exposure is significantly associated with the individual’s educational level, leisure time and firearm noise, and smoking. Incomplete adjustment for these factors leads to an overestimation of the effect of occupational noise exposure (3). Noise-induced hearing loss is usually accompanied by tinnitus, is untreatable and progresses with continuous exposure to harmful noise (4). 

One of the supposed factors predisposing a person to noise-induced hearing loss is low pigmentation and a low melanin content of the inner ear (5,6). It is already known that the embryonic origin of human melanocytes is the neural crest and that they are located in the epidermis, hair bulbs of the skin, the uveal tract, retinal pigment epithelium of the eyes, leptomeninges and the inner ear (7, 8). The presence of otic melanocytes was initially described by Alphonse Corti in 1831 (9); these cells are primarily localized throughout the stria vascularis and modiolus of the cochlea, but they also exist in the vestibular organs (8,10-12). Considering the fact that hearing is affected in systemic disorders that affect pigmented areas such as Vogt-Koyanagi and Waardenberg syndromes, melanocytes may play an important role in the inner ear (9). 

It is interesting to note that the presence of melanocytes is not limited to the peripheral auditory system. Brainstem abnormalities have been diagnosed in both animals and humans with pigment disorders; moreover, studying albino rabbits has shown that they have 24% smaller neurons in the medial superior olivary nucleus in comparison with normal animals and that the branching density of the dendrites of these cells is significantly reduced (13); this indicates that melanocytes are also present in the central auditory system. The functions of otic melanocytes are not yet completely known, they do not seem to be essential for normal hearing and these pigments are thought to have a protective role against environmental damage (14). Murillo Cuesta and colleagues (14) stated that in comparison with pigmented mice, albino mice have a higher prevalence of age-related hearing loss and poorer recovery of auditory thresholds after noise-exposure.

In 1980 Carter and colleagues conducted a study on 257 students in their third year of primary school, which aimed at investigating the correlation between eye color and susceptibility to noise-induced hearing loss. After performing audiometric tests it was seen that at 4 kHz sound frequency, hearing loss in the left ear of those children who had low pigmentation of the iris (blue or green) was higher than those with a highly pigmented iris (black or brown) (15).

The main goal of this present study was to compare firing noise-induced hearing loss in military recruits with light-colored (blonde or light brown) hair with hearing loss in those with dark-colored hair. We also aimed to evaluate the exact level of hearing loss in the same two groups following exposure to gunfire noise. 

## Materials and Methods

This was a prospective case-control study. The study population consisted of a group of military recruits in the age range of 18 to 29 years with a normal baseline audiogram, who had all taken the same two-week routine course of military education and then visited a specially designed army clinic. Among them, 57 cases including 114 ears were randomly selected for further follow up.

The research material was a specially designed questionnaire consisting of questions on demographics and hair color; occupational noise history including number of firings, weapon type and training in and use of hearing protection; and non-noise risk factors including head injury, ear disease, medications and solvent exposure. Audiometry and tympanometry studies were also used in this investigation.

The military recruits who had undertaken the two-week educational course were re-evaluated with audiologic tests 10 days after finishing the course. At this time point the aforementioned questionnaire was filled in by 62 cases. The subjects’ medical files were studied for the presence of a previous normal audiogram (taken before the educational course). Based on the specially designed questionnaire, those with a normal initial audiogram and who had been exposed to a similar number of firings (10 at least) with a similar weapon type were selected for the study. The selected military forces were then divided into two groups: dark-colored hair (black and dark brown) and light-colored hair (light brown and blond). Subsequently, their post-educational-course audiometries were compared. Among them, one who had blue eyes with dark hair and two with light brown-colored eyes were excluded from the study to eliminate the iris colour effect. Based on the data collected from the questionnaires, most of the study population had fired with a Klashinkov weapon, the most commonly used weapons following that were a Klashinkov machine gun, G3, Dushka, G3 machine gun and RPG; the cases other than Klashinkov shooters were also omitted from the study to eliminate sound variation bias. In total 57 cases were entered into the study. None of the studied military men had used self-protective equipment or were affected with any known disease. Ototoxic drug usage history was also negative in all cases.

The required study sample size with a confidence interval of 95%, (due to the undetermined level of hearing loss induced by firing noise according to the Canadian hearing-speech and language society (16) was estimated to be at least 37 cases. The collected data were analyzed by applying a paired sample t-test and ANOVA using SPSS software version 11.

As exposing human subjects to harmful noise is not ethically acceptable, the studied cases in this research had been exposed to this type of noise regardless of the researchers’ will; therefore no data existed on the received noise dosage or the level of noise received by each case. Nevertheless, the two groups were matched for the number of firings and type of weapon used. This study was approved by the ethics committee of Shahid Beheshti University of Medical Sciences and all the patients were completely informed about the study protocol and provided informed consent.

## Results

The studied cases included 20 subjects (40 ears) with light-colored hair and 37 (74 ears) with dark-colored hair. They all had a normal tympanogram and acoustic reflex. The age range of the light-colored group was 18 to 29 years with a mean age of 25.5 ± 1.8 years; all such cases were right-handed shooters. The age range of the dark-colored hair group was 18 to 28 years with a mean age of 26.5 ± 1.2 years; one case in this group was a left-handed shooter. Table 1 shows the mean hearing threshold of the right ear at 2, 3, 4, 6 and 8 kHz sound frequencies in both groups. According to Table 1, among the five tested frequencies the mean threshold of the right ear in those with light-colored hair at a frequency of 6000 Hz (29.5±17.3 dB) showed a significant difference in comparison to that of the dark-colored hair group (14.4±9.9 dB,P< 0.05); indicating that those with light-colored hair showed a higher level of right ear hearing loss in comparison to the other group at this special frequency. No remarkable difference was detected at the other investigated frequencies (Table 1).

**Table 1:** Mean and standard deviation of the right ear hearing threshold (dB HL) in the studied cases

**Frequency (kHz)**	**Hair color**	**Number of Cases**	**Mean (dB) ± SD**	**P**
2	Dark	37	7.9 ± 7.3	NS (> 0.05)
	Light	20	8.0 ± 4.8	
3	Dark	37	10.5 ± 13.0	NS (> 0.05)
	Light	20	18.5 ± 12.9	
4	Dark	37	17.6 ± 12.8	NS (> 0.05)
	Light	20	26.0 ± 18.8	
6	Dark	37	14.4 ± 9.9	S (0.008)
	Light	20	29.5 ± 17.3	
8	Dark	37	14.7 ± 13.6	NS (> 0.05)
	Light	20	22.0 ± 19.0	

NS, non-significant; S, significant; SD, standard deviation

Table 2 compares the hearing threshold of the left ear between the two groups at sound frequencies of 2, 3, 4, 6 and 8 kHz. Comparing the left-ear hearing thresholds showed that the difference in mean firing-induced hearing loss at 3 and 4 kHz was significant between the two groups (P<0.05). The mean left-ear hearing threshold in those with dark-colored hair at 3 and 4 kHz frequencies was 10 ± 9.5 and 13.8 ± 11.3 dB, respectively. The same values increased to 22 ± 17.1 and 30.5 ± 21.4 dB in the light-colored hair group (P< 0.05), respectively (Table 2).

Based on the obtained results it could be concluded that firing noise-induced damage is predictable based on a consideration of the lightness of hair color; those with light hair have a greater susceptibility when compared with those with dark hair.

**Table 2:** Mean and standard deviation of the left ear hearing threshold (dB HL) in the studied cases

**Frequency (kHz)**	**Hair color**	**Number of Cases**	**Mean (dB) ± SD**	**P**
2	Dark	37	5.8 ± 7.3	NS (> 0.05)
	Light	20	8.0 ± 4.8	
3	Dark	37	10.0 ± 9.5	S (0.027)
	Light	20	22.0 ± 17.1	
4	Dark	37	13.8 ± 11.3	S (0.013)
	Light	20	30.5 ± 21.4	
6	Dark	37	19.1 ± 14.4	NS (> 0.05)
	Light	20	23.0 ± 17.6	
8	Dark	37	15.8 ± 20.4	NS (> 0.05)
	Light	20	22.5 ± 24.4	

NS, non-significant; S, significant; SD, standard deviation

## Discussion

Permanent noise-induced hearing loss is caused by either an acoustic trauma (e.g. a brief exposure to a very intense blast-like sound) or chronic long term exposure to the loud sounds associated with a noisy occupation(17). A long-standing observation in noise-induced hearing loss has been that some ears are more susceptible to noise than others. Individually varying sensitivity to noise-induced hearing loss has been found in research in both humans and animals. Identifying individual differences among various factors has long been a focus of interest. Numerous potentially important variables having been investigated include age, gender, race, previous damage to the cochlea, efficiency of the acoustic reflex, smoking, and the influence of certain disorders such as hyperlipidemia, diabetes, and hypertension. In addition, a few factors, such as pigmentation seem to have some relationship to potentiating noise damage (17).

The present study showed that the hearing threshold of the right and left ear of the studied cases are not necessarily identical and that the left ear showed a higher level of hearing loss. It is already known that left-ear hearing loss is more probable in right-handed people (17). Asymmetrical hearing sensitivity in the right and left ear of those exposed to firing noise, especially at 3, 4 and 6 kHz frequencies, may be due to the fact that in a person who fires with the right hand, the right ear is covered by the head and shoulder and the left ear will have a greater exposure to noise (19); particularly herein where except for one case the other studied military recruits were right-handed. Temporary noise-induced hearing loss has no specific identified frequency and occurs in a wide frequency range (18), therefore it was seen that at 3, 4 and 8 kHz sound frequencies no meaningful difference existed in the mean hearing loss of the right ear. This is consistent with the results of a study by Barrenas and Lindgren (18).

Previous studies conducted by researchers (18,20) have shown that exposure to moderate noise (90–110dB) is not associated with an effect of low pigmentation on the damage caused by noise-induced hearing loss and higher noise levels have a greater impact in this respect. Due to this reason and despite our expectation, when studying guinea pigs it was revealed that both red guinea pigs and albino ones lost the same amount of hair cells when exposed to moderate noise (pure tone of 1 kHz and 105 dB SPL (Sound Pressure Level) intensity for 72 hours) (21). In the current study the hypothesis that high intensity noise (above 150 dB) has a significant association with pigmentation, was proven. Although the exact prevalence of blue eyes among the military forces is not clear, it seems that there is a remarkably lower prevalence of blue eyes compared to brown and black eyes. In the current study no cases with blue eyes were included, whereas all of the studied cases had brown or black eyes. Previous literature considering the effect of melanin on susceptibility of the inner ear to high-noise environments have focused on the person's eye and skin colors and have estimated the melanin content of the inner ear based on such indicators. To the authors’ best knowledge no similar study on the relationship of hair color and noise-induced hearing loss has been conducted. Based on our findings, the lightness level of the hair can be used as a susceptibility indicator towards firing noise-induced hearing loss in military personnel.

Regarding the responses given to the questionnaire, subjects appreciated the potential benefit of wearing hearing protectors but instructions in their proper use and education on the hazards of noise exposure were poor and the available hearing protection devices were reported to be incompatible with other gear, uncomfortable, and an impediment to communication.

## Conclusion

The obtained results indicate that a person's susceptibility to firing noise can be predicted by the lightness of their hair. In this respect those with light hair are more susceptible to firing noise-induced hearing loss in comparison with dark haired individuals. As the important role of the melanin content of the inner ear in the damage caused through high-noise environments has been proven in many studies, it is suggested that the melanin content of the inner ear of military personnel should be estimated based on the lightness of their hair, and in this way firing noise-induced hearing loss would be avoidable in susceptible cases. 

Based on our results we highly recommend that approaches to the upgrading of hearing conservation strategies and methods for strengthening the existing scheme for hearing conservation be used to further minimize such related risks. 
